# Melatonin-Pretreated Mesenchymal Stem Cells Improved Cognition in a Diabetic Murine Model

**DOI:** 10.3389/fphys.2021.628107

**Published:** 2021-03-18

**Authors:** Shaimaa Nasr Amin, Nivin Sharawy, Nashwa El Tablawy, Dalia Azmy Elberry, Mira Farouk Youssef, Ebtehal Gamal Abdelhady, Laila Ahmed Rashed, Sherif Sabry Hassan

**Affiliations:** ^1^Department of Basic Medical Sciences, Faculty of Medicine, The Hashemite University, Zarqa, Jordan; ^2^Department of Medical Physiology, Faculty of Medicine, Cairo University, Cairo, Egypt; ^3^Department of Histology, Faculty of Medicine, Cairo University, Cairo, Egypt; ^4^Department of Medical Biochemistry and Molecular Biology, Faculty of Medicine, Beni-Suef University, Beni Suef, Egypt; ^5^Department of Biochemistry, Faculty of Medicine, Cairo University, Cairo, Egypt; ^6^Department of Medical Education, School of Medicine, California University of Science and Medicine, San Bernardino, CA, United States; ^7^Department of Anatomy, Faculty of Medicine, Cairo University, Cairo, Egypt

**Keywords:** cognition, diabetes, melatonin, stem cells, synaptic plasticity

## Abstract

Diabetes mellitus (DM) is a multisystem endocrine disorder affecting the brain. Mesenchymal stem cells (MSCs) pretreated with Melatonin have been shown to increase the potency of MSCs. This work aimed to compare Melatonin, stem cells, and stem cells pretreated with Melatonin on the cognitive functions and markers of synaptic plasticity in an animal model of type I diabetes mellitus (TIDM). Thirty-six rats represented the animal model; six rats for isolation of MSCs and 30 rats were divided into five groups: control, TIDM, TIDM + Melatonin, TIDM + Stem cells, and TIDM + Stem *ex vivo* Melatonin. Functional assessment was performed with Y-maze, forced swimming test and novel object recognition. Histological and biochemical evaluation of hippocampal Neuroligin 1, Sortilin, Brain-Derived Neurotrophic Factor (BDNF), inducible nitric oxide synthase (iNOS), toll-like receptor 2 (TLR2), Tumor necrosis factor-alpha (TNF-α), and Growth Associated Protein 43 (GAP43). The TIDM group showed a significant decrease of hippocampal Neuroligin, Sortilin, and BDNF and a significant increase in iNOS, TNF-α, TLR2, and GAP43. Melatonin or stem cells groups showed improvement compared to the diabetic group but not compared to the control group. TIDM + Stem *ex vivo* Melatonin group showed a significant improvement, and some values were restored to normal. *Ex vivo* melatonin-treated stem cells had improved spatial working and object recognition memory and depression, with positive effects on glucose homeostasis, inflammatory markers levels and synaptic plasticity markers expression.

## Introduction

Diabetes mellitus (DM) is a systemic disease and may produce various adverse outcomes on the brain, such as neuronal damage, oxidative stress, decreased synaptic plasticity, glutamate neurotransmission changes, and dysfunction of astrocytes with abnormal neuronal activities (Arvanitakis et al., 2004; Son et al., 2015).

Bone marrow has two forms of stem cells: hematopoietic stem cells and mesenchymal stem cells (MSCs) (Krabbe et al., 2005). MSCs secrete cytokines and trophic factors, which activate neurogenesis, angiogenesis, and synaptogenesis that improve neural and cognitive functions (Lee et al., 2010). However, MSCs produce basal reactive oxygen species (ROS) to maintain cell proliferation and differentiation, which may cause DNA damage in MSCs and impair the normal function of MSCs (Jeong and Cho, 2016).

Melatonin is secreted by the pineal gland and has antioxidant and anti-inflammatory properties (Tordjman et al., 2017). The administration of Melatonin protects MSCs from oxidation, inflammation, apoptosis, ischemia, and aging and influences the differential activity of neural stem cells (Ma et al., 2013; Tang et al., 2014).

The neurorestorative effects of Melatonin pretreated stem cells on the brains of diabetic models and the underlying molecular mechanisms need more clarification. Therefore, this study aimed to evaluate and compare the effects of Melatonin, MSCs, and MSCs pretreated with Melatonin on cognition, behavior, and depression in an animal model of type I diabetes mellitus (TIDM) with a trial to elucidate more molecular actions and mechanisms by measurement of hippocampal markers and histopathological evaluation.

## Materials and Methods

The Ethics and Scientific Committee, Department of Physiology, Kasr Al Ainy Faculty of Medicine, Cairo University, Egypt, approved the experimental steps and research Protocol.

### Experimental Animals and Groups

Thirty-six adult male Albino rats, weighing between (120 and 150) g, were housed in cages (three rats/cage, to avoid isolation stress), exposed to normal room temperature (∼18–23°C) and humidity (40–60%) in a 12-h:12-h light-dark cycle light/dark cycles with free access to water and standard diet.

### Isolation and Propagation of BM-Derived MSCs From Rats

We used six rats for the isolation of bone marrow-derived MSCs. After the euthanasia of the rats, the marrow was isolated. Under sterile conditions, the rats’ femur and tibia were excised, and all connective tissue attached to bones was removed with special attention. Bone marrow was harvested by flushing the tibiae and femurs with Dulbecco’s modified Eagle’s medium (DMEM, GIBCO/BRL) supplemented with 10% fetal bovine medium (GIBCO/BRL). Nucleated cells were isolated with a density gradient [Ficoll/Paque (Pharmacia)] and resuspended in a complete culture medium supplemented with 1% penicillin-streptomycin (GIBCO/BRL).

Cells were incubated at 37°C in 5% humidified CO2 for 12–14 days as primary cultures or to form large colonies. When large colonies developed (80–90% confluence), cultures were washed twice with phosphate buffer saline (PBS), and cells were trypsinized with 0.25% trypsin in 1mM EDTA (GIBCO/BRL) for 5 min at 37°C. After centrifugation (at 2,400 rpm for 20 min), cells were resuspended with serum-supplemented medium and incubated in 50 cm^2^ culture flask Falcon). The resulting cultures were referred to as first-passage cultures (Alhadlaq and Mao, 2005). On day 14, the adherent colonies of cells were trypsinized and counted.

### Mesenchymal Stem Cell Surface Markers to Identify MSCs From Other Bone Marrow Cells by Flow Cytometry

**FIGURE 1:**
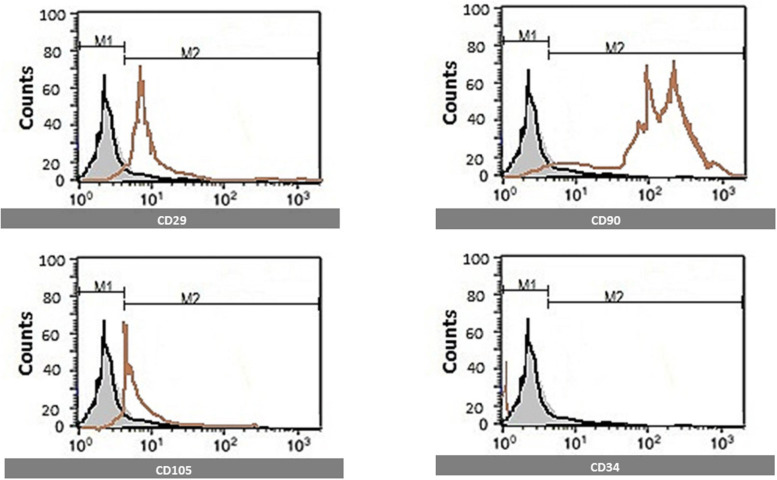
Identification of MSCs surface markers by flow cytometry. Cells are identified by flow cytometry, which was positive for CD29, CD90, and CD105 and negative for CD34.

CD29, CD90, CD105, and CD34 MSC markers were quantified using flow cytometric analysis. Florescent Activated cell sorting (FACS) analysis was used. After brief centrifugation, cells were resuspended in a wash buffer (BD Biosciences, Germany). A total of 300 ml of cell suspension was incubated with antibodies against CD29, CD105, CD34, and CD90 conjugated with Allophycocyanin (APC), Cyanine 5 (CY5), Phycoerythrin (PE), and Fluorescein isothiocyanate (FITC) dyes for 45 min at room temperature. Flow cytometry was performed on a FACS Calibur (BD Biosciences, Germany), and Cell Quest software was used for analysis. Cells are identified by flow cytometry, which was positive for CD29, CD90&CD105, and negative for CD34 (Figure 1).

MSCs cells were harvested and labeled with PKH26 fluorescent linker dye (Elberry et al., 2016).

The remaining thirty animals were divided into five groups (six rats/group):

- Control: This group was injected with the vehicle alone.
- TIDM: Diabetes was induced by a single intraperitoneal (IP) injection of STZ (50 mg/kg), which was dissolved in freshly prepared 0.1M citrate buffer (pH 4.5) (Patel and Goyal, 2011). We supplied a 5% glucose solution orally to the rats to prevent hypoglycemia during the first 24 h following the streptozocin (STZ) (Sigma-Aldrich, St. Louis, MO) administration (Patel et al., 2013). After 1 week, we measured the blood glucose levels, and rats with blood glucose levels of more than 200 mg/dl were enrolled in the study (Kaur et al., 2016).
- TIDM + MEL: This group was treated with IP injection of Melatonin (10 mg/kg/day) for 2 weeks (Negi et al., 2011), which started 4 weeks after diabetes induction.
- TIDM + STM: This group was treated with a single MSCs dose of 1 × 10^6^ per rat labeled with PKH26 in a serum-free medium by intravenous injection in rat tail vein 4 weeks after diabetes induction (Kyriakou et al., 2008).
- TIDM + STM *ex vivo* MEL: This group was treated with stem cells (after attenuation with melatonin *ex vivo*. MSCs were treated with Melatonin (5 μM) for 24 h and extensively washed with phosphate buffer and injected into the rats), 4 weeks after diabetes induction (Mias et al., 2009).

Cognitive and behavioral tests were performed twice (at the start of the work and the end of the work before euthanasia during the light phase of the light–dark cycles). Blood samples were collected (from orbital sinuses) to measure the serum fasting blood glucose level and insulin level. After the euthanasia, the brains were extracted for biochemical and histological evaluation of the hippocampus.

### Cognitive and Behavioral Assessments

The rats were acclimatized to laboratory conditions and handled daily for a week before experiments.

#### Y-Maze

A wooden Y-maze has three arms of equal size (60 cm long, 11.5 cm wide, and 25 cm high). The rats were placed at the maze center and allowed to explore the three arms for the 8-min to test spatial working memory. Parameters measured were number of arm entries, number of alternations (manually recorded), and alternation score. Any three consecutive choices of three different arms were counted as the correct choice. The alternation score was calculated by dividing the total number of alternations by the total number of choices minus 2 × 100 (Arai et al., 2001; Amin et al., 2015).

#### Novel Object Recognition Test

The rats habituated to the open field arena before being exposed to the objects; during habituation, the rats were allowed to explore an empty arena. A total of 24 h after habituation, the animals were exposed to the familiar arena with two identical objects placed at an equal distance. Each rat was placed into the open field box and exposed to the two objects for 3 min. The rats then returned to their home cage for a 1-min inter-trial interval. A total of 24 h later, the entire box was cleaned, both objects removed, and one was replaced with an identical familiar copy and one with a novel object (consistent in height and volume but are different in shape and appearance). Following the 1-min intertrial interval, rats were returned to explore the familiar and novel object in the test box for a 3-min retention trial, and the exploration time of each object and the discrimination index (DI) percentage are recorded (DI = (Novel Object Exploration Time/Total Exploration Time) – (Familiar Object Exploration Time/Total Exploration Time) × 100) (Grayson et al., 2007; Arqué et al., 2008).

#### Forced Swimming Test

The forced swimming behavioral test (FST) is used to study depressive-like behavior as reflected by the periods of immotility if the animals are subjected to the forced swimming in a cylindrical container of water from which it cannot escape. Rats were placed into a glass cylinder filled with water (depth 40 cm, 25 ± 1°C) for two successive days. On the first day, rats were placed in the water for 15 min for habituation, dried, and returned to their home cage. On the second day, rats were placed in the water for 6 min and, immobility time in the final 5 min was measured. The water was changed after every session to avoid any influence on the next rats’ behavior. Rats were monitored for the following during FST: *climbing* (upward-directed movements of the forepaws along the side of the swim chamber), *swimming* (the movement throughout the swimming chamber that also includes crossing into another quadrant), and *immobility* (no additional activity is observed other than that required to keep the rat’s head above the water) (Detke and Lucki, 1996).

### Biochemical Measurements

#### Serum Glucose and Insulin

Serum glucose levels were assayed by conventional kits supplied by (Diamond Diagnostics, Hungary). According to manufacturer instructions, insulin was assessed by an enzyme-linked immunosorbent assay (ELISA) (Linco Research, United States).

#### Gene Expression by Real-Time PCR of Hippocampal Sortilin, GAP-43, Inducible Nitric Oxide Synthase, Neuroligin 1, and Toll-Like Receptor 2

*Total RNA extraction:* Total RNA was extracted from hippocampal homogenate using RNA Isolation System (Qiagen, United States) according to manufacturer instructions. The RNA concentrations and purity were measured with an ultraviolet spectrophotometer.

*Complementary DNA (cDNA) synthesis:* The cDNA was synthesized from 1 μg RNA using SuperScript III First-Strand Synthesis System described in the manufacturer’s protocol (#K1621, Fermentas, Waltham, MA, United States).

*Real-time quantitative PCR*: Real-time PCR amplification and analysis were performed using an Applied Biosystem with software version 3.1 (StepOne^TM^, United States). The reaction contained SYBR Green Master Mix (Applied Biosystems) and gene-specific primer pairs, which were shown in Table 1 and designed with Gene Runner Software (Hasting Software, Inc., Hasting, NY) from RNA sequences from the gene bank. All primer sets had a calculated annealing temperature of 60°. Quantitative RT-PCR was performed in a 25-μl reaction volume consisting of 2X SYBR Green PCR Master Mix (Applied Biosystems), 900 nM of each primer, and 2μl of cDNA. Amplification conditions were as follows: 2 min at 50°, 10 min at 95°, 40 cycles of denaturation for 15 s, and annealing/extension at 60° for 10 min. Data from real-time assays were calculated using the v1.7 sequence detection software from PE Biosystems (Foster City, CA). Relative expression of studied gene mRNA was calculated using the comparative Ct method. All values were normalized to beta-actin, used as the control housekeeping gene, and reported as fold changes over background levels detected in the diseased groups.

**TABLE 1:** The primer sequence of the studied genes.

	**Primer sequence**
Sortilin	Forward primer 5′-CCGTCCTATCAATGTGATTAAG-3
	Reverse primer 5′-CCATATGGTATAGTCCTTCTC-3
GAP-43	Forward primer 5′-TTTCCTCTCCTGTCCTGCTC-3′
	Reverse primer 5′-TGGACTTGGGATCTTTCCTG-′3
iNOS	Forward primer 5-CCC TTC CGA AGT TTC TGG CAG CAG C-3
	Reverse primer 5-G GTT TCC GTG TTC TGA GAC TGT GGG-3
Neurogilin	Forward primer 5′-CGCAAGTGAAATCTCCTCCG-3
	Reverse primer 5′-CAATCAGCTCACGAAACTTG-3
TLR2	Forward primer 5′-GTACGCAGTGAGTGGTGCAAGT-′3
	Reverse primer 5′-GGCCGCGTCATTGTTCTC-3
Beta-actin	Forward primer 5′-GGTCGGTGTGAACGGATTTGG-3
	Reverse primer 5′-ATGTAGGCCATGAGGTCCACC-3

*GAP-43: Growth Associated Protein 43, iNOS: inducible nitric oxide synthase, TLR2: toll-like receptor 2.*

#### Tumor Necrosis Factor-Alpha and Brain-Derived Neurotrophic Factor

Tumor necrosis factor-alpha (TNF-α) and Brain-Derived Neurotrophic Factor (BDNF) were determined by ELISA kits (R&D system, United States) according to manufacturer instruction.

### Histological Methods and Detection of Homing in the Brain

Rats were euthanized by decapitation and exsanguinated. For uniform preservation and proper histological examination of the brain, intracardiac fixation perfusion was performed (Gage et al., 2012), then the brains were placed in 10% formaldehyde for 2 h then removed and placed in a new formaldehyde solution for 24 h before being dehydrated using ethanol, then cleaned in xylene and embedded in paraffin. Coronal section cuts were made with a microtome (Leica RM2025, Germany) at 5 μm thicknesses, mounted on glass slides, and stained with the hematoxylin and eosin (Bancroft, 2008).

**FIGURE 2:**
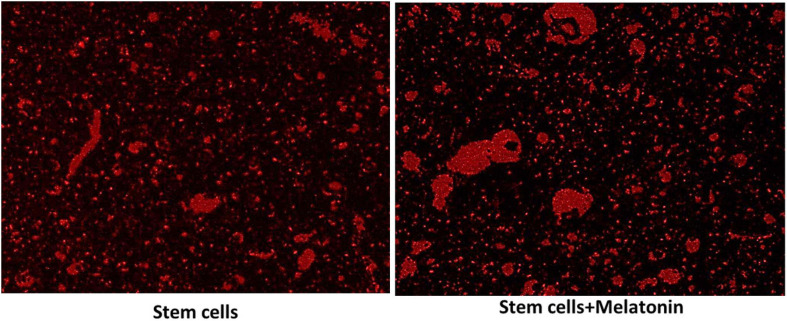
Detection of MCSs homing into the brain (×200). The red fluorescence detected MSCs groups in brain tissues, which indicate the homing of PKH26 labeled stem cells and stem cells treated with melatonin as seen more signal detected in groups with stem cells + melatonin.

For detecting cell homing, unstained sections were examined under the inverted fluorescent microscope to detect cells stained with PKH26 dye in the hippocampus (Figure 2). The number of PKH26-positive stained cells were counted from 10 non-overlapping low power field/section.

Apoptotic cells in hippocampus areas (CA1 and CA2) were quantified in 10 serial non-overlapping high-power fields for each rat.

### Statistical Analysis

Data were analyzed by the statistical package SPSS version 21(IBM Corporation, Armonk, NY) and presented as mean and standard deviation. Analysis of variance (ANOVA) with multiple comparisons *post hoc* Bonferroni was used for comparisons between groups. The Pearson correlation coefficient was used to assess the correlation between quantitative data. P values ≤ 0.05 were considered statistically significant (Altman, 2005).

## Results

### Cognitive and Behavioral Results

**FIGURE 3:**
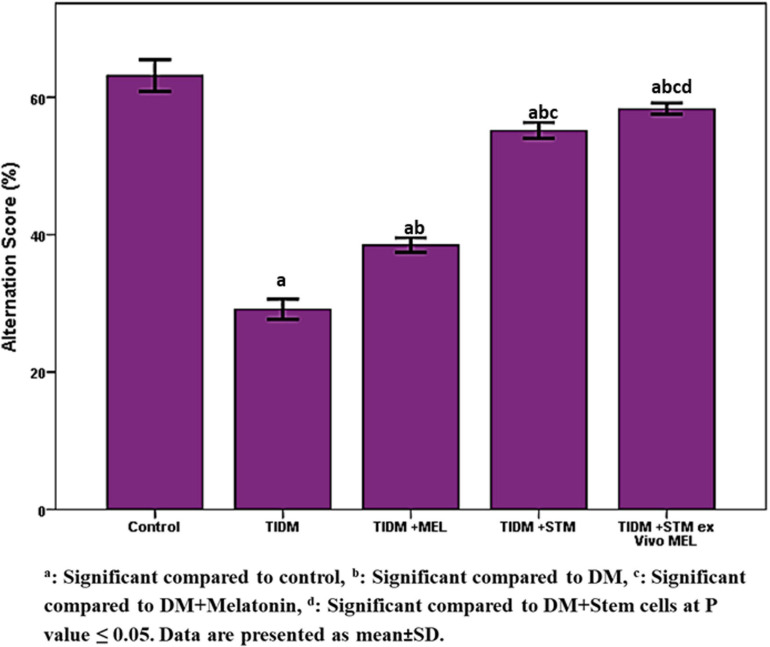
Alternation score in Y-maze task in the studied groups. The alternation score was calculated by dividing the total number of alternations by the total number of correct choices minus 2 × 100 (any three consecutive choices of three different arms were counted as the correct choice). ^a^Significant compared to control, ^b^significant compared to DM, ^c^significant compared to DM + Melatonin, ^d^significant compared to DM + stem cells at *P*-value ≤ 0.05. Data are presented as mean ± SD.

The alternation score (Figure 3) was significantly (*P* values ≤ 0.05) decreased in diabetic rats compared to the control group. The group treated with stem cells showed a significant (*P* values ≤ 0.05) increase in alternation score compared to the diabetic group and group treated with melatonin alone. The group treated with stem cells pretreated with melatonin showed a significantly (*P* values ≤ 0.05) increased alternation score compared to groups treated with stem cells or melatonin alone.

**FIGURE 4:**
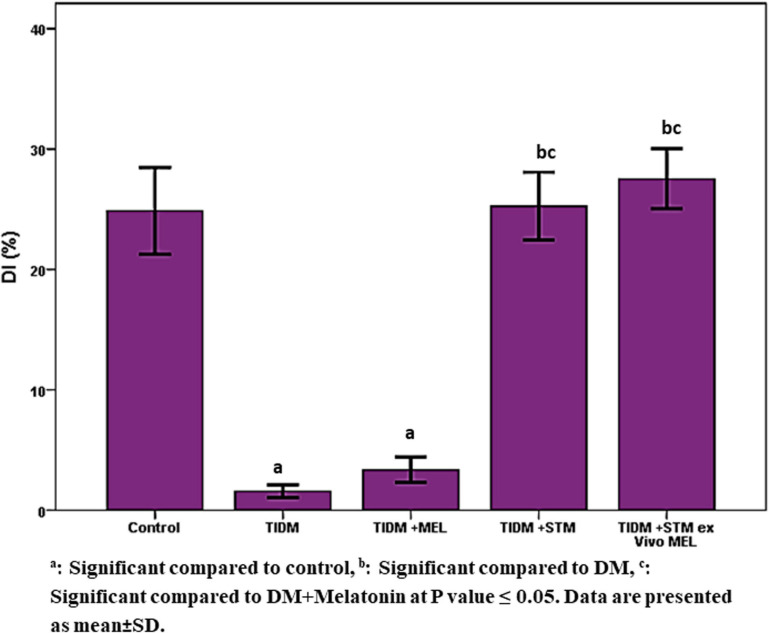
Discrimination index in novel object recognition task (NORT) in the studied groups. [DI = (Novel Object Exploration Time/Total Exploration Time) – (Familiar Object Exploration Time/Total Exploration Time) × 100]. ^a^Significant compared to control, ^b^significant compared to DM, ^c^significant compared to DM + Melatonin at *P*-value ≤ 0.05. Data are presented as mean ± SD.

Performance of the studied groups in the novel object recognition test (NORT) (Figure 4) showed that the DI at the novel object was significantly (*P* values ≤ 0.05) decreased in the diabetic group compared to the control group. Treatment with melatonin did not improve it, and the result was similar to the diabetic group. However, stem cells or stem cells pretreated with melatonin significantly (*P* values ≤ 0.05) increased DI to a similar value similar to the healthy control group.

**FIGURE 5:**
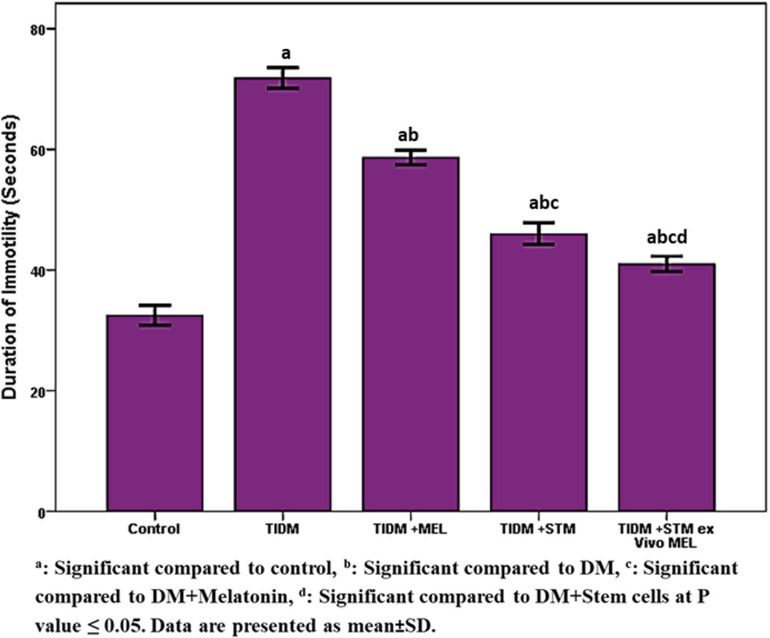
Duration of Immobility in forced swimming test (FST) in the studied groups. Immobility during FST*:* no additional activity is observed other than that required to keep the rat’s head above the water. ^a^Significant compared to control, ^b^significant compared to DM, ^c^significant compared to DM + Melatonin, ^d^significant compared to DM + stem cells at *P*-value ≤ 0.05. Data are presented as mean ± SD.

The duration of immotility in the forced swimming test (Figure 5) was significantly (*P* values ≤ 0.05) increased in the diabetic group compared to the control group. Treatment with melatonin, stem cells or stem cells pretreated with melatonin significantly (*P* values ≤ 0.05) decreased the duration of immotility compared to the diabetic group with the best improvement was in the group received stem cells pretreated with melatonin.

### Detection of MSCs Homing

As seen in Figure 2, the red fluorescence detected MSCs groups in brain tissues (subiculum and molecular layer), indicating the homing of PKH26-labeled stem cells and stem cells treated with melatonin.

Cells were characteristically found within the molecular layer and within the nerve fiber layer outside the CA zones, mainly located around capillaries, from which they supposedly migrated to reach CA. Gradually density of these cells decreased, and they were not seen within CA. A possible explanation is that they were unrecognizable within the target zones/location as they differentiated and matured.

There is more signal detected in the group with stem cells + melatonin. The cell count of the cells labeled with PKH26 in stem cells + melatonin group/field (133.57 ± 14.50) was significantly (P-value ≤ 0.05) increased compared to the stem cells group (237.14 ± 19.760).

### Biochemical Results

As shown in Table 2 for serum measurements, serum glucose was significantly increased (*P*-value ≤ 0.05) in the diabetic group. Treatment with melatonin or stem cells alone or pretreatment of stem cells attenuated by melatonin significantly decreased (*P*-value ≤ 0.05) compared to the diabetic group with the best glucose homeostasis achieved in the group treated with stem cells attenuated by melatonin.

**TABLE 2:** Serum glucose and insulin in the studied groups.

	**Control**	**TIDM**	**TIDM + Melatonin**	**TIDM + stem cells**	**TIDM + stem *ex vivo* Melatonin**
Glucose (mg/dl)	105.33 ± 7.26	280.86 ± 11.67^a^	171.13 ± 12.01^ab^	151.03 ± 6.33^abc^	143.23 ± 8.002^abc^
Insulin (ng/ml)	3.32 ± 0.18	0.77 ± 0.14^a^	1.39 ± 0.21^ab^	2.1 ± 0.004^abc^	2.19 ± 0.18^abc^

*^*a*^Significant compared to control, ^*b*^significant compared to TIDM, ^*c*^significant compared to TIDM + Melatonin, at *P*-value ≤ 0.05. Data are presented as mean ± SD.*

Serum insulin was significantly decreased in the diabetic group. Treatment with melatonin or stem cells significantly increased it compared to the diabetic group. Treatment with stem cells attenuated by melatonin significantly increased serum insulin level compared to the diabetic group and melatonin treated group but no significant difference compared to the group treated with traditional MSCs.

For the parameters measured in the hippocampus (Table 3): In the TIDM group, there was a significant (*P*-value ≤ 0.05) decrease of neuroligin, Sortilin, BDNF and a significant increase of inducible nitric oxide synthase (iNOS), TNF-α, toll-like receptor 2 (TLR2), Growth Associated Protein 43 (GAP43). Groups treated with either melatonin or stem cells showed improvement of the biochemical markers compared to the diabetic group but not compared to the control group. Groups treated with stem cells attenuated by melatonin showed significant (*P*-value ≤ 0.05) improvement in the measured parameters and values normalized to control values for neuroligin, sortilin, TLR2, and GAP43.

**TABLE 3:** Hippocampal markers in the studied groups.

	**Control**	**TIDM**	**TIDM + Melatonin**	**TIDM + stem cells**	**TIDM + stem *ex vivo* Melatonin**
Neuroligin 1 (relative expression)	1.062 ± 0.086	0.195 ± 0.039**^a^**	0.678 ± 0.1136^ab^	0.832 ± 0.1083^ab^	0.967 ± 0.1815^bc^
Sortilin (relative expression)	0.95 ± 0.076	0.34 ± 0.037**^a^**	0.57 ± 0.099^ab^	0.75 ± 0.102^abc^	0.97 ± 0.088^bcd^
BDNF (pg/ml)	120.68 ± 3.37	30.36 ± 4.75^a^	82.550 ± 5.9494^ab^	84.200 ± 6.933^ab^	108.78 ± 6.131^abcd^
iNOS (relative expression)	1.01 ± 0.043	15.50 ± 2.628^a^	8.95 ± 0.576^ab^	7.20 ± 0.429^ab^	4.70 ± 0.568^abcd^
TLR2 (relative expression)	1.198 ± 0.336	12.303 ± 2.4702^a^	6.088 ± 0.8^ab^	3.720 ± 0.4^ab^**^c^**	2.250 ± 0.288^bc^
TNF alpha (pg/ml)	31.27 ± 3.90	168.98 ± 10.88^a^	88.95 ± 3.425^ab^	67.08 ± 4.53^abc^	64.45 ± 7.302^abc^
GAP43 (relative expression)	1.07 ± 0.116	4.35 ± 0.660^a^	2.90 ± 0.548^ab^	2.49 ± 0.424^abc^	1.76 ± 0.195^bc^

*TIDM: type I diabetes mellitus, BDNF: Brain-Derived Neurotrophic Factor, iNOS: inducible nitric oxide synthase, TLR2: toll-like receptor 2, TNF alpha: Tumor necrosis factor-alpha, GAP43: Growth Associated Protein 43. ^*a*^Significant compared to control, ^*b*^significant compared to TIDM, ^*c*^significant compared to TIDM + Melatonin, ^*d*^significant compared to TIDM + stem cells at *P*-value ≤ 0.05. Data are presented as mean ± SD.*

A significant positive correlation found between number of PKH26 labeled cells/field and (indicating homing) and BDNF (*r* = 0.680; *P* = 0.015) and significant negative correlation between the number of PKH26/field and iNOS (*r* = −0.616; *P* = 0.033), TLR2 (*r* = −0.816; *P* = 0.001), and GAP43 (*r* = −0.869; *P* = 0.000).

### Histological Results

**FIGURE 6:**
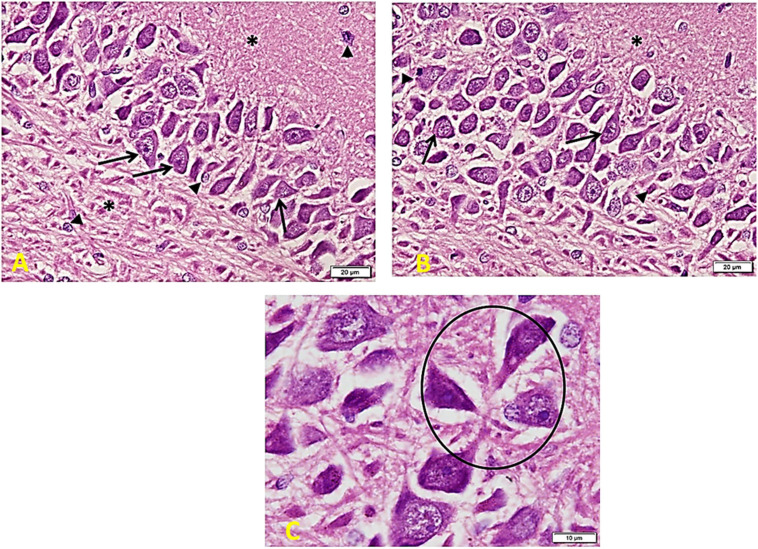
Hippocampus imaging in the control group. **(A)** A regular compact arrangement of large pyramidal cells of CA regions. Cells are nearly 3–5 layers thick. They show large central vesicular nuclei and basophilic staining (arrows) (denoting the cells’ activity). The smaller glial cells (arrowheads) can also be seen among the neurons and within the nerve fiber layers (*). Note the regular arrangement of neuronal processes in the lower part of the field (×200). **(B)** CA region of small pyramidal cells arranged in 5–8 layers. Cells are smaller in size and also show large vesicular nuclei. Glial cells (arrowheads) lie in close association to neurons (×200). **(C)** Cells are triangular in shape, with large vesicular nuclei and prominent nucleoli – note areas of connection between cells (oval area) (×400).

*In the Control group (Figure 6)*, there is a regular arrangement of large CA regions’ pyramidal cells. Cells are nearly 3–5 layers thick. They show large central vesicular nuclei and basophilic staining (arrows) (which are denoting the activity of the cells). The smaller glial cells (arrowheads) scatter among the neurons and within the nerve fiber layers (^∗^). Note the regular arrangement of neuronal processes in the lower part of the field (A). CA region of small pyramidal cells arranged in 5–8 layers. Cells are smaller in size and also show large vesicular nuclei. Glial cells (arrowheads) lie in close association with neurons (B). Cells are triangular, with large vesicular nuclei and prominent nucleoli. Note areas of connection between cells (oval area) (C).

**FIGURE 7:**
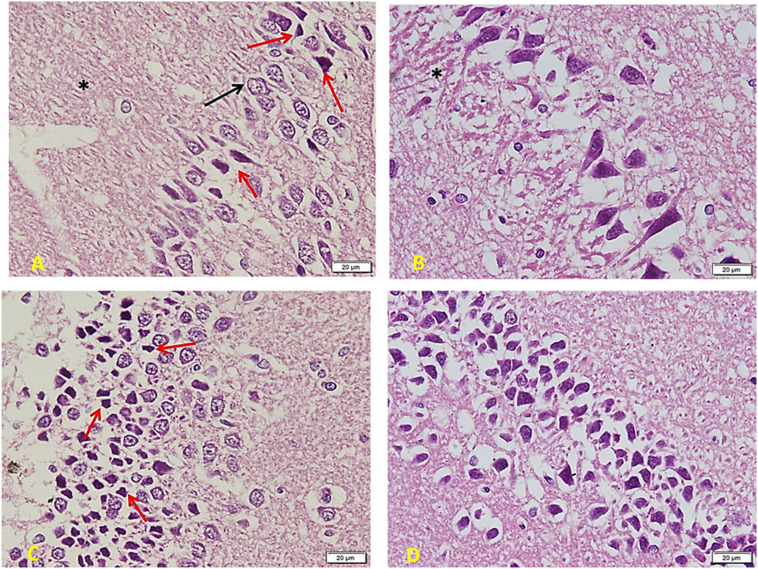
Hippocampus imaging in Diabetic group (×200). **(A)** The TIDM group shows a marked decrease in the number and crowdedness of CA regions’ pyramidal cells. Some show active vesicular nuclei (arrows) while many cells (red arrows) are shrunken with inapparent dark nuclei and surrounding empty space, denoting apoptosis. Note the decreased number of glia in between pyramidal cells as well as in between the nerve fiber layers (*). **(B)** Many areas show only one cell thickness, with wide empty spaces around cells denoting the loss of connections. Note also marked clumping and deep acidophilic staining of the neuronal processes (*). **(C)** Layer of small pyramidal cells shows marked loss of organization with many empty spaces between cells. Some are normal in appearance, but there are many apoptotic cells (red arrows) with decreased size and dark inapparent nuclei. **(D)** Other areas show widespread apoptosis (dark, small, and shrunken) and separation of cells.

*The diabetic group (Figure 7)* shows a marked decrease in the number and crowdedness of CA regions’ pyramidal cells. Some show active vesicular nuclei (arrows), while many cells (red arrows) are shrunken with inapparent dark nuclei and surrounding space, denoting apoptosis. Note a decreased glia number between pyramidal cells and between the nerve fiber layers (^∗^) (A). Many areas show only one cell thickness, with large empty spaces around cells denoting the loss of connections. Besides, a marked clumping and deep acidophilic staining of the neuronal processes can be noted (^∗^) (B). The layer of small pyramidal cells shows a marked loss of organization with many empty spaces between cells. Some are normal in appearance, but there are many apoptotic cells (red arrows) with decreased size and dark inapparent nuclei (C). Other areas show widespread apoptosis (dark, small, and shrunken) and separation of cells (D).

**FIGURE 8:**
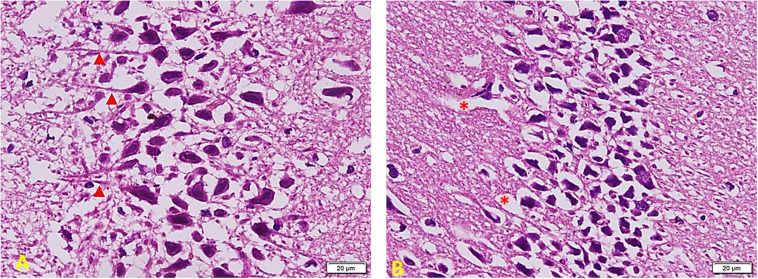
Hippocampus imaging in TIDM + Melatonin group (×200). **(A)** There is a considerable area of abnormal appearance. The pyramidal cells are large in number but markedly smaller in size. However, many cells still show standard staining and extended neuronal processes (red arrowheads) that denote cellular communication. **(B)** Most cells are markedly shrunken and separated by empty spaces around them. Other areas show clear demarcation in viable active cells and other areas of cell depletion (red *).

*Figure 8 shows the effect of melatonin treatment in diabetic rats:* a considerable area of abnormal appearance. The pyramidal cells are large in number but markedly smaller in size. However, many cells still show standard staining and extended neuronal processes (red arrowheads) that denote communication between them (A). Most cells are markedly shrunken and separated by empty spaces around them. Other areas show clear demarcation in viable active cells and other cell depletion areas (red^∗^) (B).

**FIGURE 9:**
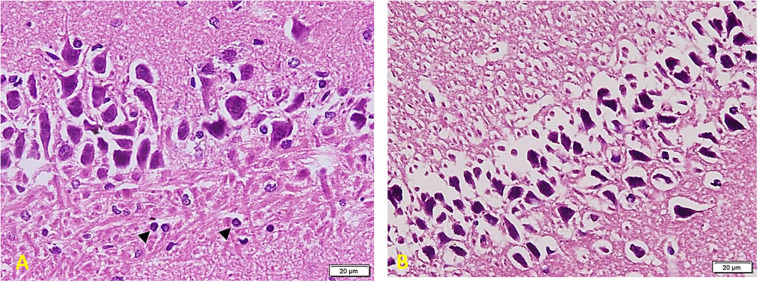
Hippocampus imaging in TIDM + MSCs group (×200). **(A)** Part of the field shows cell arrangement in nearly 3–5 layers (left side) while other areas show a marked cell depletion (right side). Cells are slightly smaller in size (compared to the control group), but the staining and shape of nuclei are similar to the control group. Note the regular arrangement of neuronal processes in the lower part of the section and the increased number of Glial cells (arrowheads). **(B)** Other areas show shrunken and separated cells similar to the DM group (most probably due to failure to improve structure).

*In Diabetic rats treated with MSC (Figure 9):* part of the field shows cell arrangement in nearly 3–5 layers (left side) while other areas show a marked cell depletion (right side). Cells are slightly smaller in size (compared to the control group), but the staining and shape of nuclei are similar to those of the control group. Note the regular arrangement of neuronal processes in the lower part of the section and the increased number of Glial cells (arrowheads) (A). Other areas show shrunken and separated cells similar to the TIDM group (most probably due to failure to improve structure) (B).

**FIGURE 10:**
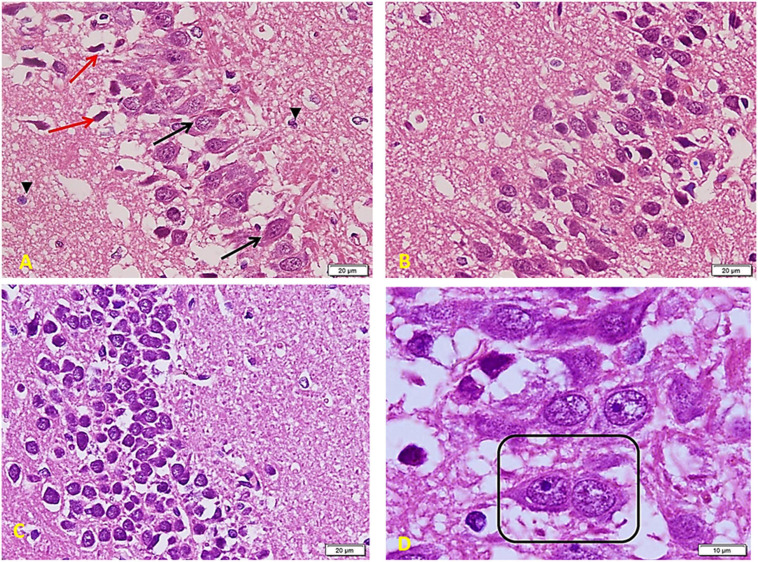
Hippocampus imaging in TIDM + Melatonin *ex vivo* MSCs group (×200). **(A)** There is a marked improvement as compared to the TIDM group. Cells are arranged in many layers but still with many empty spaces in between (as compared to the control group). Most of the cells are large and basophilic with large vesicular nuclei (arrows). A few only show apoptosis signs (red arrows) with a lack of connection to the surrounding cells. Small glial cells are distributed between neurons and between processes (arrowheads). **(B)** Layers of pyramidal cells show a large number of cells (comparable to the control group), a more compact arrangement, and less number of apoptotic cells when compared to TIDM. Most cells show large vesicular nuclei filling the basophilic cell body. The neuronal arrangement is also more homogenous than the diabetic group. **(C)** Layers of small pyramidal cells with a better arrangement and fewer spaces than the DM group. Most of the cells are viable and of average size, deep basophilic stained with large active nuclei. **(D)** Note nuclei are very close to each other (boxed area), marking a very recent cell division completion.

*In diabetic rats that received ex vivo treated stem cells with Melatonin (Figure 10)*, there is a marked improvement compared to the TIDM group. Cells are arranged in many layers but still with many empty spaces in between (as compared to the control group). Most of the cells are large and basophilic with large vesicular nuclei (arrows). Few ones only show apoptosis signs (red arrows) with a lack of connection to the surrounding cells. Small glial cells are distributed between neurons and between processes (arrowheads) (A). Layers of pyramidal cells show many cells (comparable to the control group), a more compact arrangement, and fewer apoptotic cells than TIDM. Most cells show large vesicular nuclei filling the basophilic cell body. The neuronal arrangement is also more homogenous than the diabetic group (B). Layers of small pyramidal cells with a better arrangement and fewer spaces as compared to the DM group. Most of the cells are viable and of average size, deep basophilic stained with large active nuclei (C). Note nuclei are very close to each other (boxed area), marking a very recent cell division completion (D).

The quantification of apoptotic cells in the hippocampus revealed that MSC treatment resulted in a significant decrease in apoptosis. The apoptosis quantification was more obviously reduced in the TIDM + Stem *ex vivo* Melatonin group (Table 4).

**TABLE 4:** Decreased apoptosis following melatonin-pretreated mesenchymal stem cells in hippocampal CA1 and CA3 regions of diabetic rats.

	**Control**	**TIDM**	**TIDM + Melatonin**	**TIDM + stem cells**	**TIDM + stem *ex vivo* Melatonin**
Number of apoptotic neurons in CA 1 area	2.0 ± 0.6	14.0 ± 3.3^a^	13.0 ± 0.6^a^	7.8 ± 0.7^abc^	4.8 ± 0.7^abcd^
Number of apoptotic neurons in CA 3 area	3.0 ± 0.6	28.0 ± 0.6^a^	23.6 ± 7.9^a^	14.6 ± 1.0^abc^	4.5 ± 0.8^bcd^

*TIDM: Type I Diabetes Mellitus. ^*a*^Significant compared to control, ^*b*^significant compared to TIDM, ^*c*^significant compared to TIDM + Melatonin, ^*d*^significant compared to TIDM + stem cells at *P*-value ≤ 0.05. Data are presented as mean ± SD.*

## Discussion

Diabetes is a common endocrine disease, and its negative impact affects multiple organs and systems, including the brain. The present study investigated the effect of type I diabetes in an animal model and the effect of treatment with MSCs, melatonin, and MSCs pretreated with melatonin. Functional evaluation of the studies groups involved evaluating spatial working memory, recognition memory, and depressive behavior. The outcome markers of the given treatments (which may explain the functional improvement mechanisms) included biochemical serum markers, hippocampal markers of synaptic plasticity and neurogenesis, and histological assessment of stained brain sections at the level of the hippocampus.

### Cognitive and Behavioral Tests

In the current work, we found that both spatial working memory and recognition memory were significantly impaired in the diabetic group (Figures 2, 3) as indicated by a significant decrease (*P*-value ≤ 0.05) of alternation score in the Y-maze task and time spent at the novel object in NORT. The group that received stem cells pretreated with Melatonin showed a significant increase (*P*-value ≤ 0.05) in alternation score and normalized time spent at the novel object in NORT compared to other studied groups. Moreover, Figure 4 demonstrates the significant (*P*-value ≤ 0.05) increase in immobility duration in a forced swimming test in the diabetic group, reflecting depression. The group that received Melatonin pretreated stem cells showed the best affective improvement compared to other treated groups.

The hippocampus is responsible for learning and memory through interlinked groups of circuits that are affected by chronic diseases like diabetes (Aggleton, 2012). Several studies have shown that diabetes is associated with deficits in behavioral tasks involving spatial learning and memory (Piazza et al., 2011).

Astle (2007) has concluded that depressive behavior manifestations appear only when hippocampal neurogenesis levels fall below a certain threshold, and diabetes increases the possibility of reaching this threshold.

### Histological Examination

In agreement with our results of histological assessment of the brain at the level of the hippocampus, previous studies have shown adverse effects of diabetes on the hippocampus, such as cells death, disruption of layers organization, clumping of neuronal processes, which is an indication of damage to the neurons, reduced the size of large pyramidal cells and vacuolations of Granular cell layers (Amin et al., 2013; Faheem and El Askary, 2017). Furthermore, stem cell administration by Bassiony et al. (2015) to diabetic rats has ameliorated neuron apoptosis, degeneration, and amyloid plaques formation.

### Biochemical Measurements

Owino et al. (2018) has shown that melatonin improves insulin sensitivity by activating melatonin 1 receptors (MT1) signaling at night, which modulates insulin sensitivity during the day, and this may explain insulin sensitivity improvement with melatonin reported in our study.

### For the Hippocampal Parameters

Neuroligins (NLGs) are synaptic adhesion molecules involved in synapse development and maturation and synaptic transmission through interactions with the presynaptic and postsynaptic proteins (Liu et al., 2016). NLG1 is involved in hippocampal glutamatergic synapses (Chubykin et al., 2007), long-term depression (LTD) (Dang et al., 2018), and dentate spatial pattern separation (Kesner, 2013). Similar to our results, Kim et al. (2018) have shown that stem cell therapy improved hippocampal synaptic-density loss by mediating NLG1.

Sortilin is widely expressed in the central nervous system and is involved in modifying neurotrophin activity (Hermans-Borgmeyer et al., 1999).

Brain-Derived Neurotrophic Factor is a neurotrophin with pleiotropic effects on neuronal morphology and synaptic plasticity that underlie the hippocampal circuit (Lazo et al., 2013). BDNF is initially synthesized as proneurotrophins and converted into mature neurotrophin with opposing actions of the pro and mature forms on synaptic plasticity and neuronal survival (De Vincenti et al., 2019).

In agreement with our results for hippocampal Sortilin and BDNF, Dan et al. (2020) showed reduced hippocampal expression of these markers in diabetic mice and correlated these results to cognitive function decline.

In contrast to our study, Han et al. (2019) have shown that in the diabetic model, BDNF overexpression reduced neuroinflammation in the hippocampus through the receptor for advanced glycation end products (RAGE)/nuclear factor (NF) – κB signaling pathway. However, Sun et al. (2018) have demonstrated that the reduced serum levels of BDNF were associated with an increased risk of cognitive impairment.

The change of expression of sortilin and BDNF in our study may be related to dysregulation of glucose homeostasis as hyperglycemia is related to oxidative stress, insulin resistance, and disturbances in acetylcholine homeostasis and the signaling pathway, which involves sortilin, Trk receptors, and BDNF (Morsi et al., 2019).

Mesenchymal stem cells increased expression of BDNF and IGF-1, which is vital in growth, development, neuroprotection, and repair (Dharmasaroja, 2009).

Activation of iNOS increases NO synthesis, which contributes to depression (Montezuma et al., 2012). Melatonin can decrease the effect of iNOS and NO production (Gilad et al., 1998).

Consistent with our results, several studies have shown the role of pro-inflammatory cytokines, particularly TNF-α, in diabetic neuropathy (Shi et al., 2013), depression (Simen et al., 2006), and learning and memory defects with inhibition of LTP (Alvarez et al., 2007).

Oxidative and nitrosative stress increased in diabetic patients. The excess generation of ROS and iNOS causes oxidative damage to cellular proteins, lipids, or DNA and subsequently inhibits their normal functions and disturbs homeostasis within the neuron, ultimately resulting in cell death apoptosis (Fukudome et al., 2008). Consistent with our results, El-Akabawy and El-Kholy (2014) showed an increase in the expression of iNOS and TNF-α in the hippocampus of diabetic rats.

Melatonin decreases the expression of TNF-α (Cichoz-Lach et al., 2010) and inhibits iNOS expression by inhibiting the NF-κB signaling pathway (Hao et al., 2019), and that may explain our results.

Toll-like receptors act as pattern recognition receptors and play an essential role in linking immune response with the nervous system functions, including behavior. TLRs are expressed on the surface of immune cells and astrocytes in the neurogenic niche (McKimmie et al., 2005). MSCs with a specific TLR ligand may modulate their functions and differentiation (Najar et al., 2017).

TLR2 impairs insulin-mediated brain activity (Sartorius et al., 2012) and is involved in diabetic vascular complications (Mudaliar et al., 2014). Moreover, TLR2 activation leads to the activation of the NF-κB pathway (Rolls et al., 2007), which may be another cause of increased iNOS in the diabetic group.

GAP-43 is associated with presynaptic neuronal outgrowth and neuronal plasticity and regulates the organization of presynaptic terminal and neurotransmitter release (Kosi et al., 2018). In contrast to our findings, Zhou et al. (2007) have shown a decreased hippocampal expression of GAP43 in diabetic rats.

Hung et al. (2016) have studied the effect of inflammation on GAP43 and demonstrated its upregulation by the astrocytes as a marker of reactive astrogliosis through a signaling pathway dependent on TLR-4, NF-κB, and interleukin-6. Based on increased glial cells number, which is demonstrated in this study (Figure 8), we suggest that the increased expression of GAP 43 that is demonstrated in the current work in the diabetic group is of glial origin, which denotes reactive astrogliosis, and not of neuronal origin that indicates regeneration.

Several studies have shown the effect of diabetes on reactive astrogliosis (Amin et al., 2013). In agreement with our results, He et al. (2019); have shown that MSCs reduced astrocyte reactivity in a model of hypoxic-ischemic brain injury. Furthermore, Melatonin inhibited astrogliosis in different animal models, including the diabetic model (Baydas et al., 2003).

Melatonin modulates multiple neuroprotective pathways: it enhances proteasome activity and induces the Sirtuin 1 (SIRT1) pathway (Corpas et al., 2018), provides antioxidant activity through Nuclear factor erythroid 2-related factor 2, and inhibits ER stress via prion protein (Ahmadi and Ashrafizadeh, 2019).

The administration of Melatonin improves the manipulation of MSCs *ex vivo* and *in vivo*; and protects MSCs from oxidation, inflammation, apoptosis, ischemia, and aging (Ma et al., 2013).

## Conclusion

Melatonin-treated stem cell therapy to diabetic rats had dramatically improved spatial working memory, object recognition memory, and depression with a parallel improvement in glucose homeostasis, inflammatory markers, and synaptic plasticity markers. Further studies are needed to evaluate other aspects of cognition, examine other brain areas, and evaluate other synaptic plasticity markers expression by Western blot or immunohistochemistry and not only by gene expression.

## Data Availability Statement

The raw data supporting the conclusions of this article will be made available by the authors, without undue reservation.

## Ethics Statement

The animal study was reviewed and approved by The Ethics and Scientific Committee, Department of Physiology, Kasr Al Ainy Faculty of Medicine, Cairo University, Egypt.

## Author Contributions

SA was responsible for the conceptualization, modeling, and experimental steps of cognitive and behavioral evaluation, the drafting of the manuscript, data acquisition, data analysis, and interpretation. NS shared in the experimental part and shared responsibility for the revision of the manuscript. NE and EA shared in the experimental part. DE shared in data analysis and manuscript writing. MY, LR, and SH shared in the experimental part and in writing the manuscript. All authors approved the final version of the manuscript.

## Conflict of Interest

The authors declare that the research was conducted in the absence of any commercial or financial relationships that could be construed as a potential conflict of interest.
